# Analytical approximations for the amplitude and period of a relaxation oscillator

**DOI:** 10.1186/1752-0509-3-6

**Published:** 2009-01-14

**Authors:** Carmen Kut, Vahid Golkhou, Joel S Bader

**Affiliations:** 1 Department of Biomedical Engineering, Johns Hopkins University School of Medicine, Baltimore, MD 21205, USA; 2 High-Throughput Biology Center, Johns Hopkins School of Medicine, Baltimore, MD 21205, USA; 3 Department of Biomedical Engineering, Johns Hopkins University, Baltimore, MD 21218, USA

## Abstract

**Background:**

Analysis and design of complex systems benefit from mathematically tractable models, which are often derived by approximating a nonlinear system with an effective equivalent linear system. Biological oscillators with coupled positive and negative feedback loops, termed hysteresis or relaxation oscillators, are an important class of nonlinear systems and have been the subject of comprehensive computational studies. Analytical approximations have identified criteria for sustained oscillations, but have not linked the observed period and phase to compact formulas involving underlying molecular parameters.

**Results:**

We present, to our knowledge, the first analytical expressions for the period and amplitude of a classic model for the animal circadian clock oscillator. These compact expressions are in good agreement with numerical solutions of corresponding continuous ODEs and for stochastic simulations executed at literature parameter values. The formulas are shown to be useful by permitting quick comparisons relative to a negative-feedback represillator oscillator for noise (10× less sensitive to protein decay rates), efficiency (2× more efficient), and dynamic range (30 to 60 decibel increase). The dynamic range is enhanced at its lower end by a new concentration scale defined by the crossing point of the activator and repressor, rather than from a steady-state expression level.

**Conclusion:**

Analytical expressions for oscillator dynamics provide a physical understanding for the observations from numerical simulations and suggest additional properties not readily apparent or as yet unexplored. The methods described here may be applied to other nonlinear oscillator designs and biological circuits.

## Background

Analytical expressions for dynamical systems are useful for mapping underlying parameters to observed properties. For many mechanical, electrical, and atomic systems, analysis proceeds by reducing a complicated system to tractable linear system, more often than not involving coupled harmonic oscillators. The effective analytical dynamics then provide valuable intuition even when exact results are eventually obtained using numerical methods.

For dynamics of many biological networks [1], a basic tractable component is a simple switch, with effective dynamics

(1) $$\dot{X}(t)=\beta (t)-\alpha X(t).$$

The symbol *X *represents the concentration of a molecule, for example a transcript, its encoded protein, or a specific protein modification state; *β*(*t*) is a time-dependent production rate, and *α *is a time-independent decay rate corresponding to a lifetime of *α*^-1^. The dynamics of *X *can be obtained by convolution of the input *β*(*t*) with the response function *e*^-*αt*^.

For gene regulatory networks, the input *β*(*t*) can often be modeled as an on-off toggle, *β*(*t*) = 0 in the repressed state and *β*(*t*) = *β *for full activation. This behavior arises naturally from multimeric binding of transcription factors, giving a sigmoidal Hill function as a function of transcription factor concentration.

The equilibrium behavior of this model is *X *= 0 for the repressed state and *X *= *β*/*α *for the activated state. Transients are also readily calculated. If *X *is itself a transcription factor, the relevant transient time is the delay between a change in the regulation of *X *and the subsequent change in regulation of its target genes. This introduces a second concentration, *K*, representing the concentration of *X *for half response of its target genes. For a switch from *β*(*t*) = 0 for *t *< 0 to *β*(*t*) = *β *for *t *≥ 0, the transient time is *α*^-1 ^ln [1 - *K*/(*β*/*α*)], which approaches *K*/(*β*/*α*) when the threshold *K *is a fraction of the full response *β*/*α*. For a switch from *β*(*t*) = *β *for *t *< 0 to *β *= 0 for *t *≥ 0, the transient time for decay from *β*/*α *to *K *is *α*^-1 ^ln [(*β*/*α*)/*K*], dominated by the protein lifetime *α*^-1 ^and more weakly dependent on the ratio of maximum to threshold concentration.

These basic equations can be used to predict the dynamics of bistable systems, such as metabolic switches [2]. Serial chains can give developmental progressions, such as the bacterial flagella gene network [3]. Negative feedback between simple switches can lead to bistable response, as observed in Delta-Notch signaling [4] and used to create a synthetic toggle switch [5].

Sustained oscillations can by created by coupling simple switches in a cycle, with each switch negatively regulating its successor, termed a repressilator [6]. The repressilator's amplitude and period can be estimated with good accuracy using the dynamics of each switch, giving an amplitude of *β*/*α *and a period of roughly 3*α*^-1 ^ln [(*β*/*α*)/*K*] for the three-component repressilator. Although numerical simulations are essential for a full quantitative understanding, the analytical results clearly provide intuition regarding the parameters and parameter ratios that define the oscillator behavior. For example, a stronger promoter will increase *β*, increasing the amplitude proportionally and increasing the period with much weaker logarithmic dependence. This insight can aid in understanding the differences between observed gene and protein circuits, and knowing which knobs to tweak when designing synthetic circuits. This direct connection also enables design of oscillators with desired period and amplitude, an important prerequisite for standardizing synthetic biology [7].

**Figure 1:**
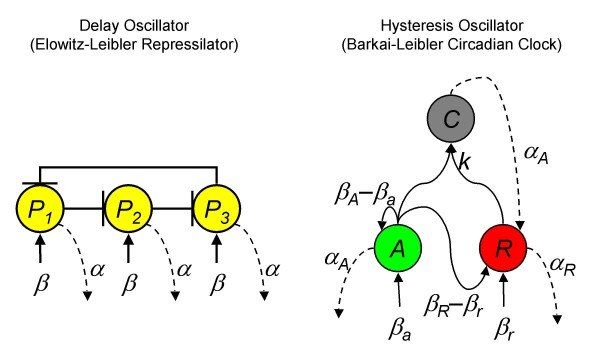
**Schematic oscillator designs. (Left) The repressilator is a delay oscillator here idealized as three symmetric repressive components, *P*_1_, *P*_2_, and *P*_3_, with identical production rates, *β*, and decay rates, *α***. (Right) The hysteresis oscillator has interlocked feedback loops involving an activator, *A*, and a repressor, *R*, which form a complex, *C*, with a bimolecular rate constant *k*. The activator and repressor have baseline production rates *β*_*a *_and *β*_*a*_, with total production rising to *β*_*A *_and *β*_*R *_when the concentration of free activator is high. The activator degrades at rate *α*_*A *_whether free or complexed; the repressor degrades at rate *α*_*R *_but only when free.

Although built in the laboratory, repressilators do not seem to be common in nature. Instead, hysteresis oscillators are thought to provide biological clocks for processes as diverse as neural signaling, basic metabolism, and development [8]. The best studied examples may be circadian clocks responsible for synchronizing living systems with day/night cycles, which are thought to have evolved independently in prokaryotes, cyanobacteria, fungi, plants, and animals [9]. A reduced model for the animal clock was introduced by Barkai and Leibler [10] (Fig. 1). Unfortunately, until now, no simple scaling rules have yet been provided oscillations arising from relaxation or hysteresis from coupled positive and negative feedback loops.

In this oscillator, an activator protein, *A*, activates transcription of both itself and a repressor, *R*. The repressor achieves its effect by forming a complex, *C*, with *A*, that does not activate transcription. Activation of *A *can generate a reservoir of *C *that serves as a continuing source of *R *in the absence of *A*, which reduces the level of *A *back to baseline. Once the *R *molecules have degraded, *A *can reactivate transcription and initiate a new cycle. These dynamics exhibit hysteresis when projected onto the *A-R *plane.

Numerical simulations indicate that hysteresis oscillators have less noisy periods than delay oscillators; in fact, noise can actually serve to prevent the clock from falling into a stable attractor, making it more robust [11]. Intracellular communication can also improve robustness [12]. Numerical models have been used to compare clocks using transcriptional versus post-translational repression elements [13]. Clocks based on the the positive-negative feedback design have been observed computationally to be more easily tuned to desired frequencies [14].

The key parameters of the hysteresis clock model are *α*_*A *_and *α*_*R*_, the decay rates of the activator and repressor proteins; *β*_*A *_and *β*_*R*_, the fully activated production rates; *β*_*a*_, the baseline production rate of the activator, and *k *is the bimolecular rate constant for $A+R\overset{k}{\underset{}{\to }}C$. The following scaling rules and approximations are developed:

1. The maximum concentration of each component is approximately proportional to *β*_*A*_/*α*_*A*_, the ratio of the production rate to the decay rate of the activator.

2. A second important concentration scale is the reset point $\sqrt{\beta_{a}/k}$ when the activator and repressor concentrations cross, rather than a nominal steady-state baseline concentration of *β*_*a*_/*α*.

3. The period is roughly divided into two phases, activation and recovery. The activation phase has duration (*β*_*A*_/*α*_*A*_)/*β*_*R*_, equivalent to the time required for repressor to titrate the equilibrium concentration of activator.

4. The recovery phase has duration $\alpha_{R}^{-1}\ln[(\beta_{A}/\alpha_{A})/\sqrt{\beta_{a}/k}]$, equivalent for the time for repressor to decay from its maximum concentration to the reset point.

This preview of the full results is accurate in the limit that production and decay rates are faster for the activator than the repressor. More accurate (but slightly more complicated) expressions are derived in the Methods. The strategy is to separate the oscillator into fast and slow subsystems that depend on the oscillator phase: during activation, *A *and *C *are fast and *R *is slow, and during recovery, *C *and *R *are fast and *A *is slow. This strategy is distinct from treatments that identify the activator as the fast subsystem throughout the entire cycle [11].

The Results show that the analytical results are accurate over a wide range of parameter space, compared with the numerical solutions to corresponding ODEs and also a stochastic simulation at the original literature values [10]. More detailed comparisons across parameters are done using ODEs alone, as the focus of this work is obtaining tractable analytics for a nonlinear system rather than investigating important known differences between ODEs and stochastic systems for small particle counts [15], or for systems where stochastic dynamics are essential for generating oscillations [16].

The value of the analytical expressions is then demonstrated through a comparison of operating characteristics: the noise, quantified as the variance of the period due to variance in production and decay rates; the cost or inverse efficiency, defined as the rate of protein production averaged over a cycle; and the dynamic range, quantified in decibels as the log-ratio of the concentration of the activating component at its maximum and minimum values. We conclude with a physical interpretation of the clock formulas and use these formulas to interpret results of computational studies.

## Results and discussion

### Hysteresis oscillator model

The protein concentrations [*A*], [*R*], and [*C*] of the activator, repressor, and complex are in units of molecules/cell and are denoted *A*, *R*, and *C *when the meaning is clear by context. The terms *A *and *R *refer only to free molecules and do not include those contained in complex *C*. The corresponding mRNA concentrations for activator and repressor are *A*_*m *_and *R*_*m*_. Continuous concentrations are assumed throughout. The mathematical model is

(2) A˙m=β′a+(β′A−β′a)An/[An+KAn]−α′AAmA˙=β″AAm−αAA−kARR˙m=β′r+(β′R−β′r)An/[An+KRn]−α′RRmR˙=β″RRm−αRR−kAR+αACC˙=kAR−αAC.

The parameters ${\beta^{\prime }}_{a}$ and ${\beta^{\prime }}_{r}$ are baseline transcriptional rates for *A*_*m *_and *R*_*m*_; ${\beta^{\prime }}_{A}$ and ${\beta^{\prime }}_{R}$ are the fully activated transcriptional rates. Transcriptional activation is represented by Hill functions with half-response at *A *= *K*_*A *_for the activator and *A *= *K*_*R *_for the repressor. The same Hill exponent *n *is used for both activator and repressor. This exponent is related to the number of activator proteins that form a transcriptional complex, and cooperative binding can result in Hill coefficients larger than 1. Although *n *= 1 was used in the original model [10], transcriptional activation in the metazoan clock is thought to be due to (hetero)dimers [9]. If binding is cooperative, *n *= 2 may be more appropriate.

Because mRNA decay rates ${\beta^{\prime }}_{A}$ and ${\beta^{\prime }}_{R}$ are fast compared to protein decay rates, min^-1 ^compared to hr^-1^, mRNA transients are brief compared to protein response. Taking the limit of fast mRNA response is equivalent to employing steady-state approximations for mRNA levels, ${\dot{A}}_{m}$ ≈ 0 and ${\dot{R}}_{m}$ ≈ 0 (see Methods).

The steady-state approximation incorporates mRNA dynamics implicitly through effective protein synthesis rates,

(3) βa=β″Aβ′a/α′AβA=β″Aβ′A/α′A

(4) βr=β″Rβ′r/α′RβR=β″Rβ′R/α′R,

and effective equations

(5) $$\begin{array}{lll} \dot{A} & = & \beta_{a}+(\beta_{A}-\beta_{a})A^{n}/[A^{n}+K_{A}^{n}]-\alpha_{A}A-kAR\\ \dot{R} & = & \beta_{r}+(\beta_{R}-\beta_{r})A^{n}/[A^{n}+K_{R}^{n}]-\alpha_{R}R-kAR+\alpha_{A}C\\ \dot{C} & = & kAR-\alpha_{A}C. \end{array}$$

For notational clarity, subscripted values of *t *are used to denote times relative to the start of the cycle, and subscripted values of *τ *refer to time intervals.

**Figure 2:**
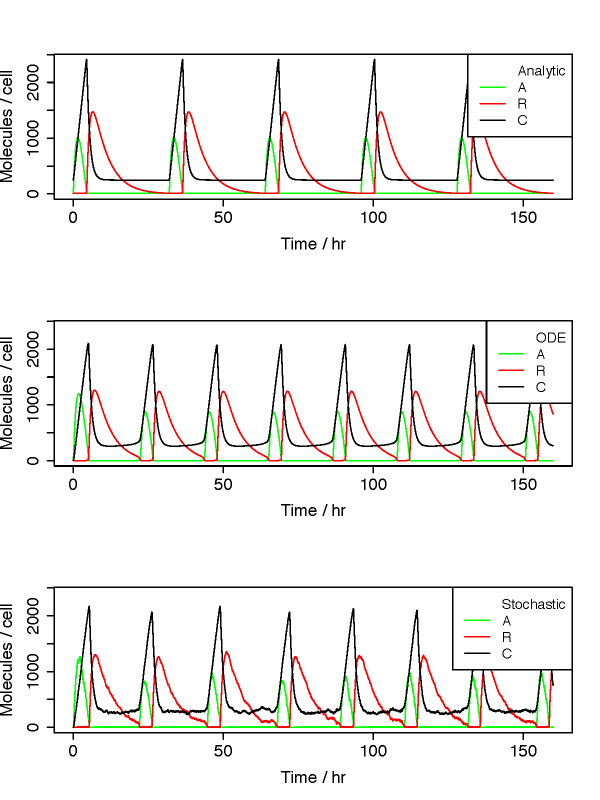
**Trajectories of activator, repressor, and complex concentrations are displayed for dynamics from analytical expressions (top panel), continuous ODEs (middle), and stochastic simulations (bottom)**. Parameter values are exactly as in Ref. [11], provided in Table 1, except with Hill coefficient = 2 to reflect cooperative dimer binding.

Central parameter values, presented in Table 1, were based on Ref. [11]. Trajectories generated using exactly these values, except with the Hill coefficient changed from 1 to 2 to reflect cooperative binding of transcription factors as dimers, are similar whether from the analytical approximate solutions to Eq. 5 derived below, the continuous-time ODE solution, or the corresponding stochastic dynamics for discrete particle numbers (Fig. 2).

**Table 1:** Circadian clock parameter values

Parameter	Units	Vilar et al. (Ref. [11])	This Work Default Value^*a*^	This Work Range
*α*_*A*_	hr^-1^	1	1	10^-1 ^.. 10^0.5^
*β*_*a*_	(mol/cell) hr^-1^	250	5	1 .. 10
*β*_*A*_	(mol/cell) hr^-1^	2500	50	10 .. 10^2^
*K*_*A*_	(mol/cell)	50	1	1 .. 10^0.3^

*α*_*R*_	hr^-1^	0.2	0.2	10^-1 ^.. 10^0.5^
*β*_*r*_	(mol/cell) hr^-1^	0.1	**0.0**	0.001 .. 0.01
*β*_*R*_	(mol/cell) hr^-1^	500	10	10^0.5 ^.. 10^1.5^
*K*_*R*_	(mol/cell)	100	2	10^0.2^..10^0.5^

*n*	Hill exponent	1	**2**	1 .. 10

*k*	(mol/cell)^-1 ^hr^-1^	2	100	20 .. 200

^*a *^Default values are based on Ref. [11] but with concentrations rescaled by a factor of 50, corresponding to half activation of *A *at one molecule per cell. The two values in bold, *β*_*r *_and *n*, differ from Ref. [11].

Unlike other studies that focus on the differences between deterministic ODEs and stochastic simulations, our aim is to develop analytical expressions that reproduce ODE behavior. For this purpose, we scaled the concentration parameters so that the concentration *K*_*A *_of *A *giving half-maximum self-activation occurs at 1 molecule per cell, compared with 50 molecules per cell from Ref. [11]. This rescaling gives a bimolecular rate constant *k *of 100 (mol/cell)^-1 ^hr^-1 ^for a pair of molecules, close to a first-principles estimate of the diffusion-limited bimolecular collision rate within the nuclear volume (see Methods). In addition to the Hill coefficient of 2 mentioned above, we also set the baseline production rate of repressor, *β*_*r*_, to zero, as opposed to the value of 0.002 that would be obtained from concentration scaling. Results using *β*_*r *_= 0 and *β*_*r *_= 0.002 are virtually identical (see Results, Comparison with ODE solutions).

### Analytical expressions for period and phase

Here we provide an overview of the method based on two assumptions:

1. Bimolecular collisions are fast compared to protein synthesis and decay rates.

2. Fast collisions between activator and repressor molecules means that the *A *+ *R *→ *C *reaction effectively goes to completion, with either *A *≈ 0 or *R *≈ 0 at all times.

The second assumption permits *A*, *R*, and *C *to be calculated from pairwise combinations that eliminate the non-linear bimolecular term. The Methods uses an expansion of the Hill functions to derive tractable dynamical equations, with summary analytical expressions provided in Table 2. The main results are sketched here using a simplified logic approximation, replacing the Hill functions with step functions [1]. These results are accurate when *A *passes quickly through its threshold value, which occurs for much of parameter space.

**Table 2:** Analytical results for oscillator period and phase

Times	Hysteresis^*a*^	Delay^*b*^
*τ*_1 _(*A *> *R*)	*n *= 1: (*β*_*A*_/*α*_*A *_*β*_*R*_) + *K*_*A*_/*β*_*A *_+(*K*_*A*_/*β*_*A*_) ln(*K*_*A*_/$\sqrt{\beta_{a}/k}$)	*α*^-1 ^ln [(*β*_1 _- *β*_0_)/(*β*_1 _- *αK*)]
	*n *≠ 1: (*β*_*A*_/*α*_*A *_*β*_*R*_) + *K*_*A*_/*β*_*A*_+ [*β*_*A*_(*n*- 1)]^-1 ^$K_{A}^{n}$(*β*_*a*_/*k*)^1-*n*^	
*τ*_2 _(*A *<*R*)	*α*_*A *_= *α*_*R*_: *α*^-1 ^ln [(*β*_*A*_/*α*)/$\sqrt{\beta_{a}/k}$]	*α*^-1 ^ln [(*β*_1 _- *αK *- *β*_0_)/(*αK *- *β*_0_)]
	*α*_*A *_≠ *α*_*R*_: $\alpha_{\min}^{-1}$ ln [(*β*_*A*_/|*α*_*A *_- *α*_*R*_|)/$\sqrt{\beta_{a}/k}$]	
*τ*_tot_	*τ*_1 _+ *τ*_2_	3(*τ*_1 _+ *τ*_2_)
*τ*_tot_, limiting	*α*^-1 ^[*β*_*A*_/*β*_*R *_+ ln(*β*_*A*_/*α *$\sqrt{\beta_{a}/k}$)]	3*α*^-1 ^ln [*β*_1_/*αK*]

Concentrations		
*A*_max_	(*β*_*A*_/*α*_*A*_)- (*β*_*R*_/*α*_*A*_) ln(*β*_*A*_/*β*_*R*_)	(*β*_1_/*α*) - *K*
*R*_max_	*α*_*A *_= *α*_*R*_: [(*β*_*A *_- *β*_*a*_)/*α*-*K*_*A*_]/*e*	
	$\alpha_{A}\neq \alpha_{R}:(\beta_{A}/\alpha_{A}){\left(\alpha_{R}/\alpha_{A}\right)}^{\alpha_{R}/\alpha_{A}}$	
*C*_max_	*β*_*A*_/*α*_*A*_	
*A*_min_	*β*_*a*_/(*α*_*A *_+ *kR*_max_)	*β*_0_/*α*
*A*_min_, limiting	*β*_*a*_/(*kβ*_*A*_/*αe*)	*β*_0_/*α*

^*a *^Limiting expressions for the hysteresis oscillator are for *α*_*A *_= *α*_*R *_= *α*, *β*_*A *_> *β*_*R*_, *β*_*A *_> *αK*_*A*_.

^*b *^Limiting expressions for the delay oscillator are for *β*_1_/*α *> *K *> *β*_0_/*α*.

The start of the cycle, *t *= 0, is defined to occur when *A *and *R *are both low with *A *increasing just past *R*. From the dynamics of *A*, $\dot{A}$ ≈ *β*_*a *_- *α*_*A*_*A *- *kAR*, and the nullcline $\dot{A}$ = 0 crosses *A *= *R *at the value $\sqrt{\beta_{a}/k+{\left(\alpha_{A}/2k\right)}^{2}}$. In the limit of a fast bimolecular reaction, $k>\alpha_{A}^{2}/4\beta_{a}$, the crossing point is at *A *= *R *= $\sqrt{\beta_{a}/k}$. The first phase of dynamics ('activation phase') ends when *A *has risen to its maximum and then returned to a low value, with *C *high and *R *still small. The end of this first phase, with duration *τ*_1_, is defined when *C *is at its maximum. In the second phase ('recovery phase'), *C *declines to a baseline value and *R *rises and falls. The end of the second phase, with duration *τ*_2_, is defined when *R *has just crossed below *A*.

During the activation phase,

(6) $$\begin{array}{lll} \left(d/dt)(A+C\right) & \approx & \beta_{a}+(\beta_{A}-\beta_{a})\Theta (A-K_{A})-\alpha_{A}(A+C)\\ \left(d/dt)(R+C\right) & \approx & \beta_{r}+(\beta_{R}-\beta_{r})\Theta (A-K_{R}), \end{array}$$

where Θ(*u*) is the unit step function. Starting from *A *= *R *= $\sqrt{\beta_{a}/k}$, A rises quickly to *K*_*A*_. The time required is approximately *K*_*A*_/*β*_*a *_(see Methods for a more accurate expression). The subsequent dynamics are

(7) $$\begin{array}{lll} A+C & \approx & \beta_{A}/\alpha_{A}+[(\beta_{a}-\beta_{A})/\alpha_{A}]\exp(-\alpha_{A}t)\\ R+C & \approx & \beta_{R}[t-(K_{R}-K_{A})/\beta_{A}], \end{array}$$

neglecting *β*_*r *_relative to *β*_*R*_. Since *R *≈ 0 in the activation phase, *R *+ *C *≈ *C*, and (*A *+ *C*) - (*R *+ *C*) > *K*_*A *_is required to maintain activation. Assuming that *A *+ *C *is close to its asymptotic value of *β*_*A*_/*α*_*A *_at this point implies *R *+ *C *= *β*_*A*_/*α*_*A *_- *K*_*A*_, or

(8) *t *= (*β*_*A *_- *α*_*A*_*K*_*A*_)/*α*_*A *_*β*_*R *_+ (*K*_*R *_- *K*_*A*_)/*β*_*A*_

for the elapsed time.

There is another brief transient in which *A *decays to 0, described using the combinations

(9) $$\begin{array}{lll} \left(d/dt)(C+R\right) & \approx & -\alpha_{R}R\approx 0\approx \dot{C}\\ \left(d/dt)(A-R\right) & \approx & \beta_{a}-\alpha_{A}(A+C)-\alpha_{R}R\approx \beta_{a}-\beta_{A}\approx \dot{A}. \end{array}$$

The second equation uses *C *≈ *β*_*A*_/*α*_*A *_during this interval. The time for this transient is approximately *K*_*A*_/(*β*_*A *_- *β*_0_).

The total time for the activation phase is therefore

(10) *τ*_1 _= (*β*_*A *_- *α*_*A*_*K*_*A*_)/*α*_*A *_*β*_*R *_+ (*K*_*R *_- *K*_*A*_)/*β*_*A *_+ *K*_*A*_/(*β*_*A *_- *β*_*a*_) ≈ (*β*_*A*_/*α*_*A*_)/*β*_*R*_

This value can be rationalized as the amount of time required for enough repressor to be produced, at rate *β*_*R*_, to neutralize the total amount of activator both free and in complex, *β*_*A*_/*α*_*A*_.

At the start of the recovery phase, the complex is at its maximum concentration of approximately (*β*_*A*_/*α*_*A*_) - *K*_*A *_≈ *β*_*A*_/*α*_*A*_. Here *A *≈ 0 and it is convenient to examine the combinations *C *+ *A *and *R *– *A *with

(11) $$\begin{array}{c} (d/dt)(C+A)=\beta_{a}-\alpha_{A}(C+A)\approx \beta_{a}-\alpha_{A}C\approx \dot{C}\\ (d/dt)(R-A)=-\beta_{a}+\alpha_{A}(C+A)-\alpha_{R}R\approx \dot{R}. \end{array}$$

For *α*_*A *_≠ *α*_*R*_, these equations give the dynamics

(12) $$\begin{array}{lll} C(t) & = & \beta_{a}/\alpha_{a}+[(\beta_{A}-\beta_{a})/\alpha_{A}]\exp(-\alpha_{A}t)\\ R(t) & = & [\beta_{A}/(\alpha_{A}-\alpha_{R})]\cdot [\exp(-\alpha_{R}t)-\exp(-\alpha_{A}t)], \end{array}$$

which continue until the concentration of *R *dips below *A *to trigger a new cycle. The concentrations cross at $\sqrt{\beta_{a}/k}$, and the duration of the recovery phase is

(13) $$\tau_{2}={\left[\min(\alpha_{A},\alpha_{R})\right]}^{-1}\ln[(\beta_{A}/|\alpha_{A}-\alpha_{R}|)/\sqrt{\beta_{a}/k}].$$

### Comparison with the ODE solutions

**Figure 3:**
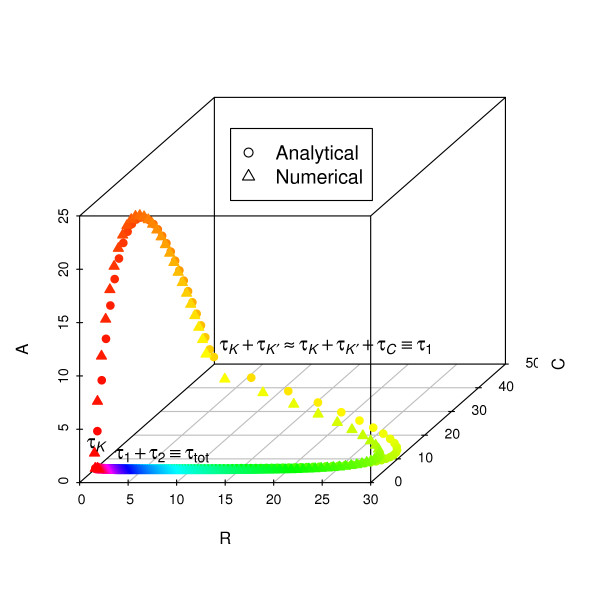
**Limit cycle from analytical expressions (circles) and numerical calculations (triangles) are displayed in three dimensions**. Parameter values are from Table 1. Points are spaced at 200 equal time increments along the trajectory, and colors of points represent time progression in ROYGBIV order. The total period is 27.4 hr from the numerical ODE solution and 32.0 hr from the analytical expression.

**Figure 4:**
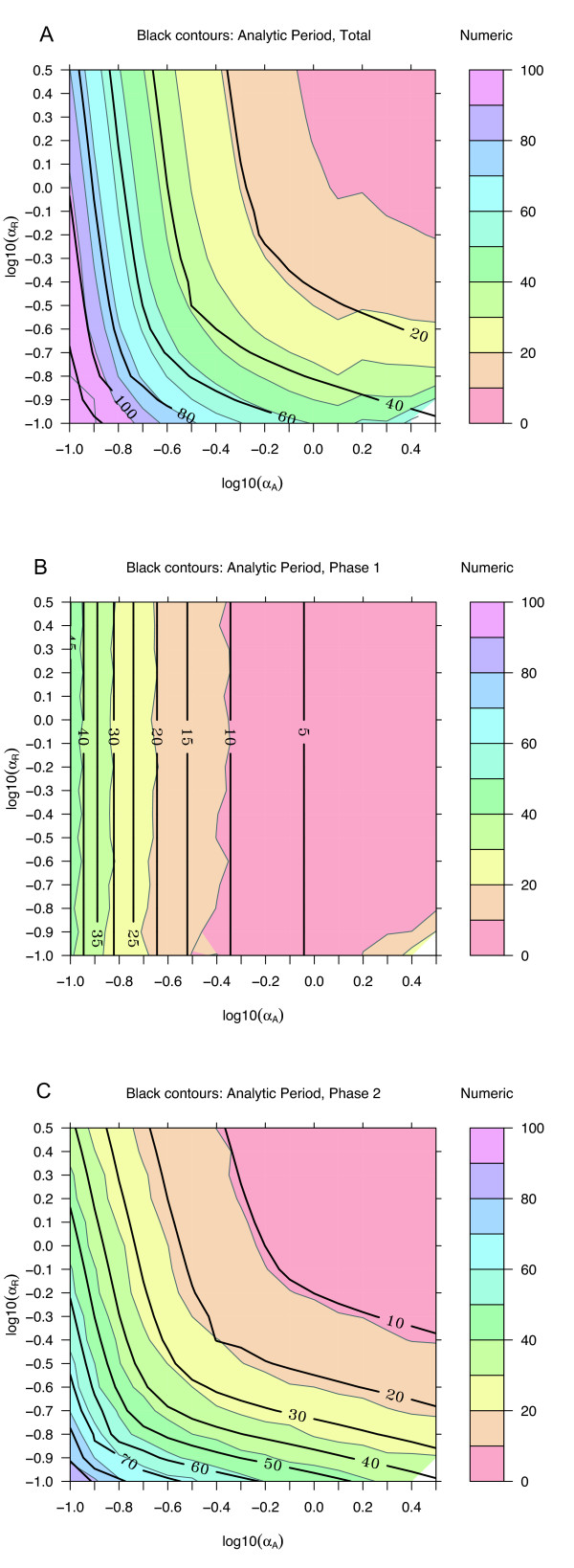
**Analytic and numeric periods for *α*_*A *_and *α*_*R*_**. Other parameter values are set to defaults from Table 1. The background is colored according to periods calculated from numerical solutions of the ODE model, and black contour lines are from analytical expressions in Table 2. **(A) **Full period, *τ*_tot_. **(B) **Activation phase, *τ*_1_. **(C) **Recovery phase, *τ*_2_. In this and subsequent figures, contour lines are estimated from values calculated on a grid indicated by light background lines. A finer grid would yield smoother contours. The general agreement of the colored contours (ODEs) and the thick contour lines (analytic) indicates an accurate model for the dependence of the period on specific parameters. For example, the time *τ*_1 _for the activation phase depends only on *α*_*A *_and not *α*_*R*_, while the recovery phase depends on both. As the degradation rates decrease, the period increases rapidly. Decreasing the degradation rates further can eliminate oscillations.

**Figure 5:**
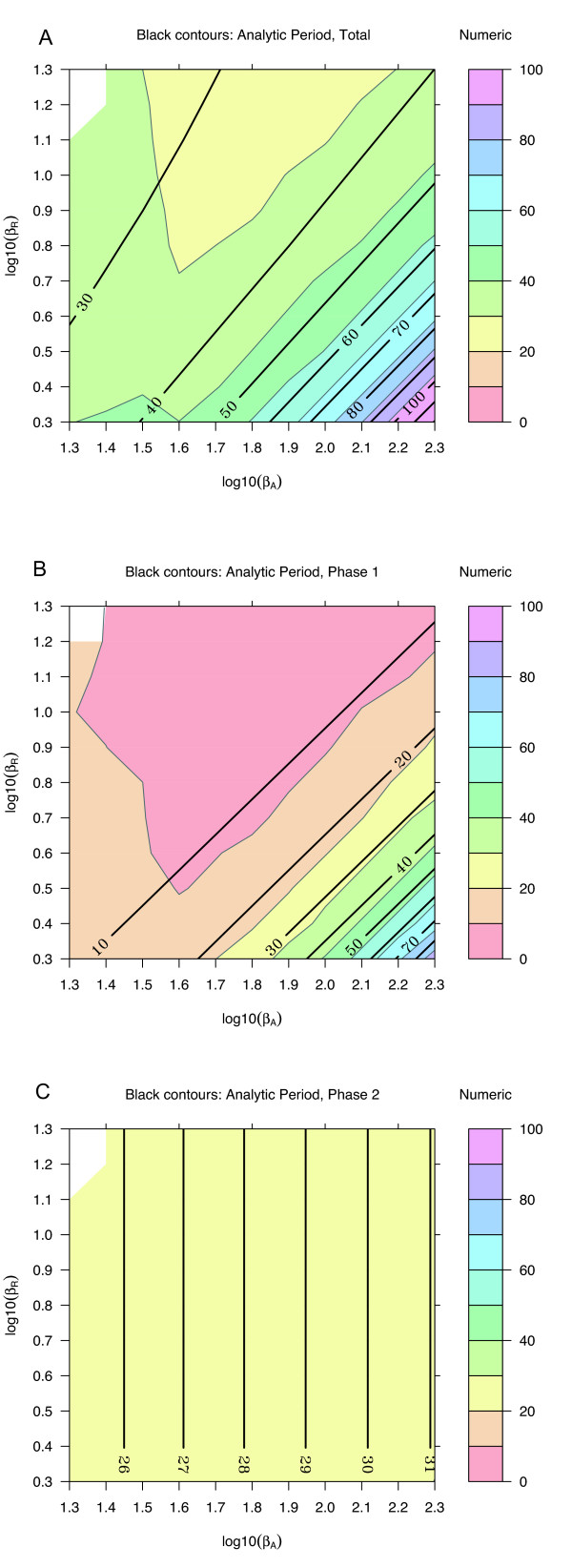
**Analytic and numeric periods for *β*_*A *_and *β*_*R*_**. Other parameter values are from Table 1, except that *β*_*a *_is set to 0.1*β*_*A*_. Plots are as in Fig. 4. The dependence on production rates is primarily in the activation phase, not the recovery phase.

**Figure 6:**
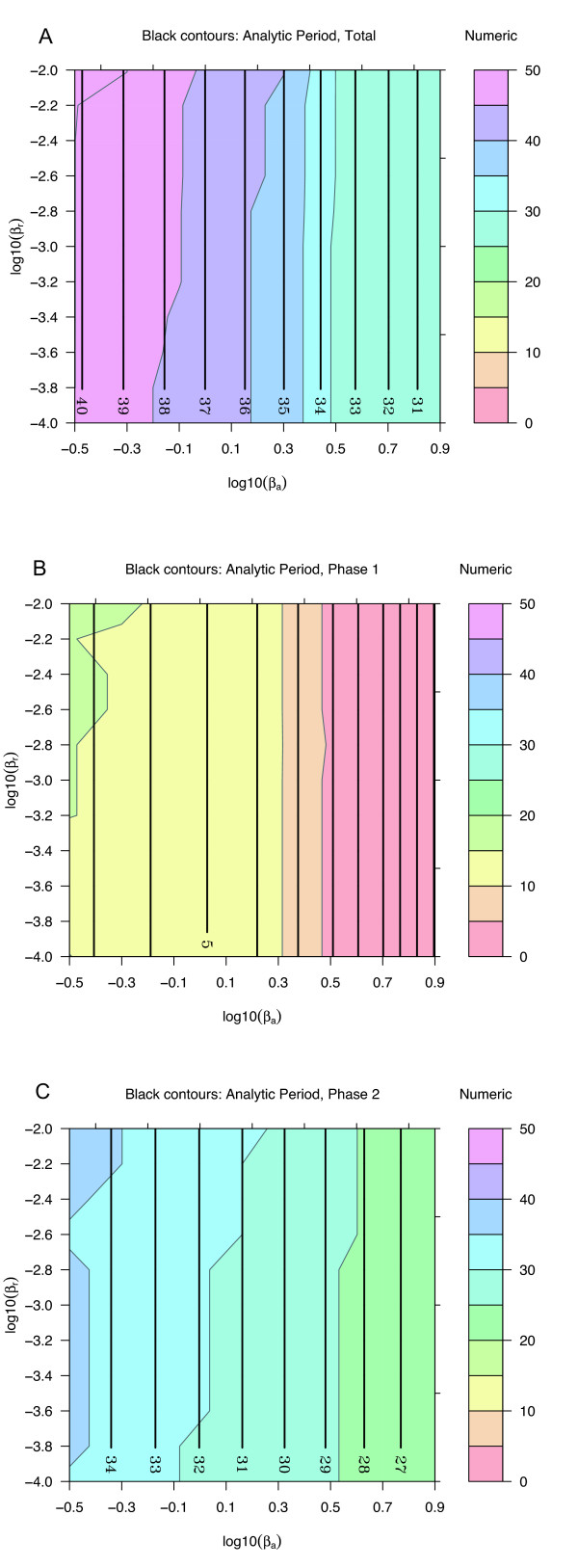
**Analytic and numeric periods for *β*_*a *_and *β*_*r*_**. Plots are as in Fig. 4. The period depends primarily on *β*_*a *_and is insensitive to *β*_*r*_.

A three-dimensional visualization of the dynamics (Fig. 3) demonstrates that the analytical expressions are in excellent agreement with numerical results from ODEs. For a more complete examination of agreement across parameter space, parameters representing decay rates (Fig. 4), activated production rates (Fig. 5), and baseline production rates (Fig. 6) were scanned over an order of magnitude. The period and the time for the individual phases are compared with numerical ODE results using 4D contour plots [17,18].

Decay rates have a strong influence on the period (Fig. 4). The activation phase depends almost entirely on *α*_*A*_, with virtually no dependence on *α*_*R*_; the recovery phase depends on the smaller of the two. The parameter space searched is symmetric about *α*_*A *_= *α*_*R*_, with *α*_*A*_/*α*_*R *_ranging from 1/30 to 30. Robust oscillations are still observed even when *α*_*R *_>> *α*_*A*_, demonstrating that oscillations do not require that *A *is the faster subsystem.

The dependence of the full period on the activated production rates scales roughly as *β*_*A*_/*β*_*R *_(Fig. 5). Most of this dependence arises from the activation phase. For the recovery phase, both the analytical and the numerical estimates suggest very little effect. This result is consistent with a low level of activator during the recovery phase. The baseline production rate of the activator does affect the time for the recovery phase, as well as the activation phase (Fig. 6). The production rate of the repressor is generally taken to be low in clock models, and over two orders of magnitude has virtually no effect on the dynamics.

### Delay oscillator

The first well-known engineered biological clock was a delay oscillator termed the repressilator [6]. Equations for a standard simplified continuous, symmetric three-component model are presented (see Methods), again using the approximation that mRNA levels decay faster than protein levels. Repressilator dynamics, periods, and amplitudes are reviewed in the Methods and included in Table 2.

In the comparisons that follow for noise (defined by variance in the period), efficiency, and dynamic range, it is necessary to introduce a correspondence between parameters of the delay oscillator and hysteresis oscillator. We assume that production rates, variance in production rates, and decay rates are equivalent in the two systems.

### Variance of the period

The noise in the oscillator period is analyzed here through the variance, Var(*τ*_tot_), providing an analytical route to sensitivity analysis of robustness [19]. Assuming that production events of *A *and *R *are correlated but uncorrelated with decay events, and that both *A *and *R *decay at the same rate *α*, this variance is

(14) $$\begin{array}{lll} \text{Var}(\tau_{\text{tot}}) & = & {\left(\partial \tau_{\text{tot}}/\partial \beta_{A}\right)}^{2}\text{Var}(\beta_{A})+{\left(\partial \tau_{\text{tot}}/\partial \beta_{B}\right)}^{2}\text{Var}(\beta_{B})+\\ & & (\partial \tau_{\text{tot}}/\partial \beta_{A})(\partial \tau_{\text{tot}}/\partial \beta_{B})\text{Cov}(\beta_{A},\beta_{B})+{\left(\partial \tau_{\text{tot}}/\partial \alpha \right)}^{2}\text{Var}(\alpha ). \end{array}$$

Using the limiting form for the hysteresis oscillator period, Table 2,

(15) $$\begin{array}{lll} \text{Var}(\tau_{\text{tot}}) & = & \alpha^{-2}[{\left(1+\beta_{A}/\beta_{R}\right)}^{2}\text{Var}(\beta_{A})/\beta_{A}^{2}+{\left(\beta_{A}/\beta_{R}\right)}^{2}\text{Var}(\beta_{R})/\beta_{R}^{2}\\ & & -2(\beta_{A}/\beta_{R})(1+\beta_{A}/\beta_{R})\text{Cov}(\beta_{A},\beta_{R})/\beta_{A}\beta_{R}]\\ & & +\alpha^{-2}\text{Var}(\beta_{a})/4\beta_{a}^{2}+{\left[\tau_{\text{tot}}+\alpha^{-1}\right]}^{2}\text{Var}(\alpha )/\alpha^{2}. \end{array}$$

To simplify this expression, we designated the correlation between *β*_*A *_and *β*_*R *_as *r *and assume that the coefficient of variation is roughly uniform for each component,

(16) $$\begin{array}{l} \text{Var}(\beta_{A})/\beta_{A}^{2}\approx \text{Var}(\beta_{R})/\beta_{R}^{2}\approx \text{Var}(\beta_{a})/\beta_{a}^{2}\approx \sigma^{2}:\\ \;\begin{array}{lll} \text{Var}(\tau_{\text{tot}}) & = & \left(\sigma^{2}/\alpha^{2})[(5/4)+2(\beta_{A}/\beta_{R})(1+\beta_{A}/\beta_{R})(1-r)\right]\\ & & +{\left[\tau_{\text{tot}}+\alpha^{-1}\right]}^{2}\text{Var}(\alpha )/\alpha^{2} \end{array} \end{array}$$

The corresponding variance for the limiting form of the delay oscillator period is

(17) $$\text{Var}(\tau_{\text{tot}})=(9/\alpha^{2})\text{Var}(\beta_{1})/\beta_{1}^{2}+{\left[\tau_{\text{tot}}+3\alpha^{-1}\right]}^{2}\text{Var}(\alpha )/\alpha^{2}$$

For purposes of comparison, we assume that $\text{Var}(\beta_{1})/\beta_{1}^{2}=\sigma^{2}$ as well, and that the decay rate *α *is also the same for the hysteresis and delay designs.

Consider separately the variance due to transcriptional noise, scaling as *σ*^2^, and variance due to decay noise, scaling as Var(*α*). The variance due to transcriptional noise will be smaller for the hysteresis oscillator than the delay oscillator when

(18) *r *≥ 1 – 31/[8(*β*_*A*_/*β*_*R*_)(1 + *β*_*A*_/*β*_*R*_)].

The parameters in Table 1 suggest *β*_*A*_/*β*_*R *_≈ 5, and noise reduction for the hysteresis oscillator requires a rather large correlation in transcription rates, *r *≥ 7/8. A smaller ratio gives a smaller correlation required for noise reduction. For example, *β*_*A*_/*β*_*R *_= 2 gives *r *≥ 1/3. Correlation arises naturally because production rates of both the activator and the repressor depend on the concentration of free activator. For the true biological system, correlation would also arise from fluctuations in the concentrations of polymerase and ribosomes.

Variation in the period due to decay noise is always larger for the delay oscillator compared to the hysteresis oscillator. Assuming a period of 24 hours and a protein lifetime of 0.5 to 5 hours, the delay oscillator has 8% to 80% higher variance in its period than the hysteresis oscillator.

### Efficiency

The efficiency of an oscillator is defined here as the inverse of its power requirements, where power is the rate of protein production averaged over a period. For the hysteresis oscillator, activator and represser molecules are produced at rates *β*_*A *_and *β*_*R *_during the activation phase, and are not produced during the recovery phase, yielding the power requirement (*β*_*A *_+ *β*_*R*_)*τ*_1_/*τ*_tot_. For the delay oscillator, the synthesis rate during phase 1 is *β*_0 _+ 2*β*_1_, and the synthesis rate during phase 2 is 2*β*_0 _+ *β*_1_, with power requirement *β*_0 _+ *β*_1 _+ (*β*_1_*τ*_1 _+ *β*_0_*τ*_2_)/*τ*_tot_.

Assuming that baseline synthesis rates are small relative to activated synthesis rates and *β*_*R*_/*β*_*A *_is no greater than 0.5 suggests these costs:

(19) $$\begin{array}{cc} \text{Hysteresis}: & (3/2)\beta_{A}\tau_{1}/\tau_{\text{tot}}\\ \text{Delay}: & \beta_{1}. \end{array}$$

If the activated production rates are similar, *β*_1 _≈ *β*_*A*_, then the hysteresis oscillator will have a cost of (3/2)*τ*_1_/*τ*_tot _relative to the delay oscillator, giving a cost advantage when *τ*_1_/*τ*_tot _< 2/3. Using typical parameters, the activation phase is faster than the recovery phase, with *τ*_1_/*τ*_tot _≈ 1/3. This gives the hysteresis oscillator a two-fold cost reduction, or equivalently a two-fold efficiency increase relative to the delay oscillator.

### Dynamic range

To be functional, an oscillator must couple to other biological components. The most straightforward coupling is for the activator molecule to serve as a transcription factor for output elements. These elements may have varying binding affinities for the activator, and it may therefore be advantageous for the activator to have a large dynamic range during a cycle. The dynamic range is quantified as decibels (dB) as 10 log_10_(*A*_max_/*A*_min_).

Using the limiting forms from Table 2, the dynamic ranges of the oscillators are

(20) $$\begin{array}{cc} \text{Hysteresis}: & 10\log_{10}(\beta_{A}/\beta_{a})+10\log_{10}(k\beta_{A}/e\alpha^{2})\\ \text{Delay}: & 10\log_{10}(\beta_{1}/\beta_{0}) \end{array}$$

For the delay oscillator, the ratio of activated to baseline production rates is typically a factor of 10 to 100, yielding a 10 to 20 dB dynamic range. The hysteresis oscillator has a similar contribution of 10 dB from the ratio *β*_*A*_/*β*_*a*_. The hysteresis oscillator has an additional contribution, however, because the minimum concentration of *A *is much lower than the conventional steady-state baseline *β*_*a*_/*α*. Again using typical values, *kβ*_*A*_/*eα*^2 ^≈ 7000 to 8000, and the effect is a boost of about 40 dB to the dynamic range.

## Conclusion

This work provides a physical interpretation for the period and dynamic range of a model for a hysteresis oscillator. The period has a first phase whose duration, approximately (*β*_*A*_/*α*_*A*_)/*β*_*R*_, can be interpreted as the time required to synthesize sufficient repressor molecules at rate *β*_*R *_to titrate an equilibrium concentration of activator molecules, *β*_*A*_/*α*_*A*_. The second phase has duration approximately equal to $\alpha_{R}^{-1}\ln[(\beta_{A}/\alpha_{A})/\sqrt{\beta_{a}/k}]$. This has the familiar form of a protein lifetime, *α*^-1^, multiplied by the log-ratio of an initial concentration to a final concentration. The initial concentration, *β*_*A*_/*α*_*A*_, corresponds to the same equilibrium concentration as before. The final concentration, $\sqrt{\beta_{a}/k}$, is the value when the activator and repressor concentrations cross.

It is intriguing that the critical step triggering the start of a new phase is the crossing of the activator and repressor concentrations. In the context of gene expression, predictors based on crossing of mRNA abundances have been remarkably powerful for classification problems [20]. The results generated here for a particular nonlinear system show that such crossings can be important in marking the transition between distinct states.

The signal that oscillations are not supported is that the time for the second phase of the cycle, *τ*_2_, becomes long. This is apparent in the contour plots showing the time for each phase. For example, in Fig. 4, when the decay constants become small, the period becomes rapidly larger. In this region, both *A *and *R *are small, and *C *is close to the value *β*_*a*_/*α*_*A*_. An expansion of the dynamical equations in this region could provide and analytically tractable expression for stability analysis.

Our results agree with numerical simulations that have found the hysteresis design to be more robust with respect to noise, but permit the ability to ascribe variance independently to production and decay sources. The hysteresis oscillator period is estimated to be roughly ten times more robust to fluctuations in decay rates. Reduction of noise from production rates requires positive correlations of at least 35% between activator and repressor production fluctuations. This observation is interesting because the hysteresis model explicitly couples the synthesis of these components during a single phase of the cycle, whereas the delay oscillator produces components continuously throughout the cycle. Same-time production of activator and repressor molecules should naturally introduce correlations in production rates because both depend on the same fluctuating concentration of activator molecules for transcriptional activation.

Our results also provide an intuitive explanation for a recent observation that coupled positive-negative feedback oscillators can cover a wider frequency spectrum than pure negative feedback oscillators [14]. For the period of the negative feedback oscillator considered here, the protein production rate appears inside a logarithm, giving it only weak influence on the period. For the hysteresis oscillator, however, protein production rates appear to linear order, with a much stronger ability to influence the period. Moreover, activator and repressor production rates appear as a ratio, permitting greater leverage for changing the period.

The analytical expressions also permit easy examination of other oscillator properties. Sustaining oscillations requires a cost that can be measured in the biomolecules that must be synthesized and then degraded over the course of a period. We estimate that the energetic cost of the hysteresis oscillator is about half that of delay oscillator. Our results suggest that efficiency may be an important oscillator property; to our knowledge, it has yet to be studied in computational models.

**Figure 7:**
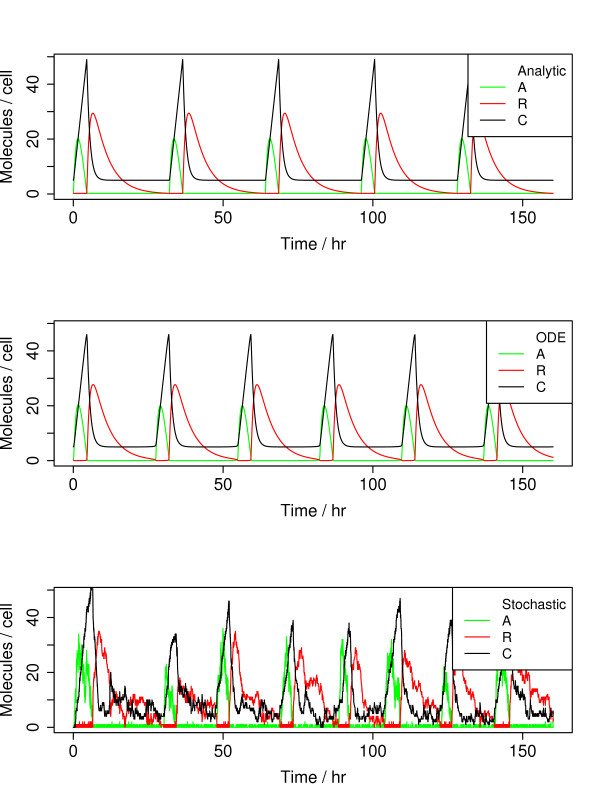
**Trajectories of activator, repressor, and complex concentrations are displayed for dynamics from analytical expressions (top panel), continuous ODEs (middle), and stochastic simulations (bottom)**. Parameter values are the default values from Table 1, essentially identical to those in Ref. [11] but with Hill coefficient = 2 and concentrations reduced by 50×.

The output of an oscillator should have a large dynamic range to maximize its ability to couple to output systems. While the dynamic range of a delay oscillator is 10 to 20 dB, the dynamic range of a hysteresis oscillator with similar transcription and decay rates is 50 to 60 dB, an impressive gain. The large dynamic range arises from a state where all concentrations are close to 0. This does not necessarily make stochastic behavior important, however: the relevant count is the number of particles required to activate transcription, *K*_*A *_or *K*_*R*_, rather than the small value $\sqrt{\beta_{a}/k}$ marking the start of a new cycle. As seen in Fig. 2 with *K*_*A *_= 50, trajectories from ODEs, analytical approximations, and stochastic simulations are quite similar. When stochastic simulations are run using parameters scaled by a factor of 50 to give *K*_*A *_= 1, however, the period is shorter and more variable than continuous ODEs or the analytical formulas (Fig. 7). For the analytical and ODE trajectories, the only difference is a 50× scaling in the output amplitudes.

In summary, these results provide a direct connection between parameters and observed properties of a circadian clock model. By showing how period and amplitude scale with parameters, these results help explain results observed in numerical simulations and suggest oscillator efficiency as an area where additional computational analysis may be valuable. The analysis strategy is to convert a nonlinear system into a series of linear systems connected at their boundaries, with a key transition marked by the crossing of activator and repressor concentrations. While the analysis here is for a particular clock model, the analysis strategy is general and should be applicable to other nonlinear biological systems.

## Methods

### Parameter values

All concentration parameters have units (number of molecules per cell), and all time units are (hours) or (per hour) for rates. A summary of default parameter values for the core circadian oscillator, based on Ref. [11], is provided (Table 1). The default parameters are generally consistent with full production response with a few activator proteins per cell, activated mRNA production rates of about one per five minutes, mRNA lifetimes of about 10 minutes, and translation rates of 5 proteins per transcript per hour.

The parameter *K*_*A*_, with units of molecules per cell, provides a concentration scale. Concentration and production-rate parameters from Ref. [11] were reduced by a factor of 50 to give *K*_*A *_= 1, corresponding to half activation at one transcription factor protein per cell. The product of the bimolecular rate constant *k *and a concentration yields a rate. Since concentrations were reduced by a factor of 50, the bimolecular rate constant was increased by a factor of 50 to yield an identical effective rate, maintaining exactly the same balance between bimolecular collision rates and protein decay rates.

Other than the concentration scaling, two other changes were made relative to Ref. [11]. First, the baseline repressor production rate was set to 0 mol/hr, compared to 0.1 mol/hr previously, or 0.002 mol/hr after the 50× concentration scaling. Second, the Hill exponent was set to 2 rather than 1, reflecting that the transcription factors in this system may bind cooperatively as dimers rather than monomers [9].

Several other parameter sets for more complete models of the circadian clock in Drosophila and mammalian systems are available [21-25]. These parameter sets are quite different, and rather than attempting to reconcile them we scanned parameters over a range of values.

A first-principles route to estimating the value of the bimolecular rate constant is to calculate a diffusion-limited reaction rate for proteins. The bimolecular rate constant for two proteins with diffusion constants *D*_1 _and *D*_2 _and effective radius *h *is

(21) *k *= 4*π*(*D*_1 _+ *D*_2_)*h*/*V*,

where *V *corresponds to a spherical volume in which diffusion occurs [26], which we take as the nuclear diameter. Typical diffusion constants for proteins are in the range of 10^-6 ^to 10^-7 ^cm^2^/sec. Using a nuclear diameter of roughly 7 *μ*m (volume = 1.8 × 10^-16 ^m^3^) and an effective protein radius of 1 nm gives a bimolecular rate constant *k *~150 to 165/(molecule-hr) when *A *and *R *have units (molecules)/(nuclear volume).

We followed the Barkai-Leibler model in assuming that the activator molecule can be degraded while free and in a complex, while the repressor can only be degraded when free and is protected in the complex. We also assumed, as in the original model, that the activator decay rate is the same for free and bound molecules.

### Justification of steady-state approximations for mRNA

Consider a protein *X *with mRNA *X*_*m *_with dynamics

(22) $$\begin{array}{c} {\dot{X}}_{m}(t)=\beta^{\prime }(t)-\alpha^{\prime }X_{m}\\ \dot{X}(t)=\beta^{\prime \prime }X_{m}-\alpha^{\prime \prime }X, \end{array}$$

where *β'*(*t*) is an mRNA synthesis rate that has explicit time dependence, for example due to fluctuating concentrations of a transcription factor. The protein synthesis rate, *β*"*X*_*m*_, has time dependence through the mRNA concentration *X*_*m *_with a constant coefficient *β"*. Using the notation that $\tilde{f}$(*s*) is the Laplace transform of *f*(*t*),

(23) X˜m(s)=(s+α′)−1[Xm(0)+β˜′(s)]X˜(s)=(s+α″)−1X(0)+β″(s+α′)−1(s+α″)−1[Xm(0)+β˜′(x)]

The transfer function relating protein output to mRNA production input is *β"*(*s *+ *α'*)^-1^(*s *+ *α"*)^-1^. When mRNA decay rates are fast compared to protein decay rates, the ratio *α"*/*α' *can be used as a small expansion parameter, yielding

(24) $$\tilde{X}(s)={\left(s+\alpha^{\prime \prime }\right)}^{-1}X(0)+(\beta^{\prime \prime }/\alpha^{\prime }){\left(s+\alpha^{\prime \prime }\right)}^{-1}[1-(s/\alpha^{\prime })+O{\left(s/\alpha^{\prime }\right)}^{2}][X_{m}(0)={\tilde{\beta }}^{\prime }(x)].$$

The notation *O*(...) indicates asymptotic order. Transforming back to the time domain yields

(25) $$\dot{X}(t)=(\beta^{\prime \prime }/\alpha^{\prime })\beta^{\prime }(t)-\alpha^{\prime \prime }X(t)+O(\alpha^{\prime \prime }/\alpha^{\prime })$$

with effective initial conditions

(26) *X*_eff_(0) = *X*(0) + (*β"*/*α'*)*X*_*m*_(0) + *O*(*α"*/*α'*).

In the model presented here, protein and mRNA concentrations are both close to 0 at time 0, and the offset to *X*(0) due to mRNA may be ignored.

### ODE calculations

Solutions to ODEs were obtained with R [27] using the interface to lsoda [28,29].

### Stochastic simulations

Stochastic simulations were performed using the Gillespie stochastic simulation algorithm [30,31] as implemented in R by the GillespieSSA package [32]. Simulations with a maximum particle count below 1000 used the direct method. Simulations with a maximum count of 1000 or more were accelerated using the binomial tau-leap method [33,34]. Six equations define the stochastic dynamics:

(27) $$\begin{array}{ccc} A & \overset{\beta_{a}+(\beta_{A}-\beta_{a}){\left(A/K_{A}\right)}^{n}/[1+{\left(A/K_{A}\right)}^{n}]}{\to } & A+1\\ A & \overset{\alpha_{A}A}{\to } & A-1\\ R & \overset{\beta_{r}+(\beta_{R}-\beta_{r}){\left(A/K_{R}\right)}^{n}/[1+{\left(A/K_{R}\right)}^{n}]}{\to } & R+1\\ R & \overset{\alpha_{R}R}{\to } & R-1\\ \left(A,R,C\right) & \overset{kAR}{\to } & \left(A-1,R-1,C+1\right)\\ \left(R,C\right) & \overset{\alpha_{A}C}{\to } & \left(R+1,C-1\right) \end{array}$$

Separating fast and slow subsystems can improve the performance of stochastic simulations [35].

### Analytical approximations

The analytical approximations are motivated by the timescale separation between fast diffusion-limited bimolecular collisions between *A *and *R*, and the remaining slow timescales in the system. When the concentrations *A *and *R *are each 1 molecule/cell, for example, and *k *is the diffusion-limited rate constant 100/(molecule × hr), the formation rate of *C *is $\dot{C}$ = 100 molecules/hr. In comparison, the maximum production rates of *A *and *R *are expected to be only 10 to 20 molecules/hr for typical parameters, and decay rates are slower still.

With this timescale separation, the bimolecular reaction effectively goes to completion: all available molecules of *A *and *R *are paired into complexes, until either *A *≈ 0 or *R *≈ 0. Depending on whether *A *or *R *is limiting, this leads to the approximation that $\dot{A}$ ≈ 0 or $\dot{R}$ ≈ 0. These approximations lead to analytical expressions, as detailed below.

### Recovery from time 0 to full activation at time *t*_*K*_

The start of the cycle, time 0, is defined when *A *and *R *are small, with *A *just crossing above *R*, and $\dot{C}$ = *kAR *- *α*_*A*_*C *≈ 0. In this regime, (*d*/*dt*)(*C *+ *A*) = *β*_*a *_- *α*_*A*_(*A *+ *C*) + (*β*_*A *_- *β*_*a*_)*A*^*n*^/[*A*^*n *^+ $K_{A}^{n}$], which is approximately equal to *β*_*a *_- *α*_*A*_(*A *+ *C*) for *A *<<*K*_*A*_. At the end of the previous cycle, the sum *A *+ *C *therefore approached an equilibrium value of *β*_*a*_/*α*_*A *_with transient time on the order of $\alpha_{A}^{-1}$. As *A *is small, the concentration *C *at time 0 is approximately *β*_*a*_/*α*_*A*_. Using $\dot{C}$ ≈ 0 and *A *= *R*,

(28) $$X\equiv A(0)=R(0)=\sqrt{\beta_{a}/k}$$

defines the crossing value *X*.

As A becomes activated but is still below *K*_*A *_and *K*_*R*_, the Hill functions are approximated to yield the equations

(29) $$\begin{array}{lll} \dot{A} & = & \beta_{a}+(\beta_{A}-\beta_{a}){\left(A/K_{A}\right)}^{n}-\alpha_{A}A-kAR\\ \dot{R} & = & \beta_{r}+(\beta_{R}-\beta_{r}){\left(A/K_{R}\right)}^{n}-\alpha_{R}R-kAR+\alpha_{A}C\\ \dot{C} & = & kAR-\alpha_{A}C. \end{array}$$

During this phase, *A *is much smaller than *K*_*A *_and *K*_*R*_, $\dot{R}$ is small, and *α*_*R*_*R *and *β*_*r *_are small relative to activated transcription of *R*, yielding the approximate dynamics

(30) kAR=(βR−βr)(A/KR)n+αAC≈(βR−βr)(A/KR)n+βa,andA˙=[(βA−βa)/KAn−(βR−βr)/KRn]An−αAA≈BXAn,withβX≡(βA−βa)/KAn−(βR−βr)/KRn.

Recovery of *A *implies $\dot{A}$ > 0 and *β*_*X *_> 0. The decay term *α*_*A*_*A *is assumed to be small compared to the production rate, which will be seen in the results to be a good approximation.

The dynamics of Eq. 30 continue until the turning point of the Hill function, which occurs at *A *≈ *K*_*A*_.

Terming *t*_*K *_as the time when *A *= *K*_*A *_in this regime, Eq. 30 is integrated implicitly to yield

(31) t(A)=∫XAdA[βXAn]−1=[βX(n−1)]−1[X−n+1−A−n+1],tK=[BX(n−1)]−1[X1−n−KA1−n],A(t)=[X1−n−(n−1)βXt]1/(1−n).

In the case that *n *= 1, it is possible to retain the term *α*_*A*_*A *in the analytical solution, and the corresponding equations are

(32) $$\begin{array}{c} t_{K}={\left(\beta_{X}-\alpha_{A}\right)}^{-1}\ln(K_{A}/X)\\ A(t)=X\exp[(\beta_{X}-\alpha_{A})t]. \end{array}$$

With typical parameters, *β*_*X *_≈ *β*_*A*_/*K*_*A *_> *α*_*A*_, and *α*_*A *_can be ignored as with the *n *> 1 case. This approximation ignores the very short transient in which *A *is between *K*_*A *_and *K*_*R*_. As will be seen from the results, very little error is made.

### The activator makes a round trip

The next analytical regime begins with *A *= *K*_*A *_and rising rapidly, *C *≈ *β*_*a*_/*α*_*A*_, and *R *≈ 0. Here full transcriptional activation is a good approximation, *A *>> *K*_*A *_and *A *>> *K*_*R*_, and the Hill functions become 1. The dynamics for *R *are $\dot{R}$ = *β*_*R *_- *α*_*R*_*R *- *kAR *+ *α*_*A*_*C*. Since $\dot{R}$ and *R *are both small, *kAR *≈ *β*_*R *_+ *α*_*A*_*C*. The dynamics for *C *are therefore $\dot{C}$ = *β*_*R*_, or

(33) *C*(*t*) = (*β*_*a*_/*α*_*A*_) + *β*_*R*_(*t *- *t*_*K*_).

An interpretation of this equation is that every new molecule of *R*, produced at rate *β*_*R*_, is converted immediately to a complex, adding to the baseline amount of complex at time *t*_*K*_.

The trajectory for *A *using Eq. 33 is

(34) A˙=βA−αAA−βR−αAC=(βA−βa−βR)−αAA−αAβR(t−tK)A(t)=KAexp⁡[−αA(t−tK)]+[(βA−βa)/αA]⋅{1−exp⁡[−αA(t−tK)]}−βR(t−tK).

The time when *A *reaches its maximum and the maximum value are

(35) tA=tK+(1/αA)ln⁡[(βA−βa−αAKA)/βn]Amax⁡=(βA−βa−βR)/αA−(βR/αA)ln⁡[(βA−βa−αAKA)/βR].

These equations continue to hold until *A *returns to value *K*_*A*_. The time *t'*_*K *_when this occurs is obtained from Eq. 34 using the approximation that the exponential transient has decayed and that *β*_*r *_is negligible,

(36) t′K=tK+(βA−βa−αAKA)/αAβR,andC(t′K)=(βA/αA)−KA≡Cmax⁡

from Eq. 33. The complex will be seen to have obtained its maximum value *C*_max _at this time.

Once *A *falls below the threshold *K*_*A*_, it continues to fall rapidly and the Hill functions are approximately 0. The conditions that $\dot{R}$ ≈ 0 and *R *≈ 0 yield *kAR *= *α*_*A*_*C*, giving $\dot{C}$ ≈ 0 as well. The dynamics for *A *in this regime are

(37) $$\begin{array}{c} \dot{A}=\beta_{a}-\alpha_{A}A-\beta_{A}+\alpha_{A}K_{A}\\ A(t)=K_{A}+[(\beta_{A}-\beta_{a})/\alpha_{A}]\cdot \{[\exp(-\alpha_{A}(t-{t^{\prime }}_{k})]-1\}. \end{array}$$

These dynamics continue until *A *= 0, at which point *C *is still equal to *C*_max_. Defining this time as *t*_*C*_,

(38) $$t_{C}={t^{\prime }}_{K}-(1/\alpha_{A})\ln[1-\alpha_{A}K_{A}/(\beta_{A}-\beta_{a})]\approx {t^{\prime }}_{K}+K_{A}/(\beta_{A}-\beta_{a}).$$

### The repressor makes a round trip

In this phase of the cycle, *A *becomes the slow subsystem because *A *and hence $\dot{A}$ are small, while *R *and *C *change more rapidly. The approximations $\dot{A}$ = 0 with *α*_*A*_*A *= 0 yield *kAR *= *β*_*a*_, and

(39) $$\begin{array}{l} \dot{C}=\beta_{a}-\alpha_{A}C\\ \dot{R}=\beta_{r}+\alpha_{A}C-\alpha_{R}R-\beta_{a}. \end{array}$$

The trajectories are

(40) $$\begin{array}{lll} C(t) & = & \beta_{a}/\alpha_{A}+[(\beta_{A}-\beta_{a})/\alpha_{A}-K_{A}]\exp[-\alpha_{A}(t-t_{C})]\\ R(t) & = & \left[(\beta_{A}-\beta_{a}-\alpha_{A}K_{A})/(\alpha_{A}-\alpha_{R})]\cdot \{\exp[-\alpha_{R}(t-t_{C})]-\exp[-\alpha_{A}(t-t_{C})]\right\}\\ & & +(\beta_{r}/\alpha_{R})\cdot \{1-\exp[-\alpha_{R}(t-t_{C})]\},\text{ or} \end{array}$$

(41) $$\begin{array}{lll} R(t) & = & \left(\beta_{A}-\beta_{a}-\alpha_{A}K_{A})\cdot (t-t_{C})\cdot \exp[-\alpha (t-t_{C})\right]\\ & & +(\beta_{r}/\alpha )\cdot \{1-\exp[-\alpha (t-t_{C})]\} \end{array}$$

in the case that *α*_*A *_= *α*_*R *_= *α*.

Ignoring the small contribution from the baseline production rate *β*_*r*_, the time *t*_*R *_for the maximum of *R *and its maximum value are

(42) tR=tC+[1/(αA−αR)]ln⁡(αA/αR),Rmax⁡=[(βA−βa)/αA−KA]⋅(αR/αA)αR/(αA−αR); ortR=tC+α−1,Rmax⁡=[(βA−βa)/αA−KA]⋅e−1

in the case that *α*_*A *_= *α*_*R *_= *α*.

An improved approximation for *A *in this region, again from $\dot{A}$ = 0, is *A*(*t*) ≈ *β*_*a*_/[*α*_*A *_+ *kR*(*t*)]. According to this expression, *A *has its minimum value when *R *is at its maximum. These dynamics continue until *R *= *X *= $\sqrt{\beta_{a}/k}$, at which point *R *≈ *A *and the cycle restarts. For convenience, let *α*_min _= min(*α*_*A*_, *α*_*R*_) and Δ*α *= |*α*_*A *_- *α*_*R*_|. Again ignoring the small contribution from *β*_*r*_, the crossing time *t*_*X *_is calculated as

(43) $$\begin{array}{lll} t_{X} & = & t_{C}+\alpha_{\min}^{-1}\ln\{[(\beta_{A}-\beta_{a}-\alpha_{A}K_{A})/\Delta \alpha \sqrt{\beta_{a}/k}]\}\\ & & +\alpha_{\min}^{-1}\ln\{1-{\left[\Delta \alpha \sqrt{\beta_{a}/k}/(\beta_{A}-\beta_{a}-\alpha_{A}K_{A})\right]}^{\Delta \alpha /\alpha_{\min}}\}. \end{array}$$

The final term in *t*_*X *_is a perturbative estimate of the correction due to the faster transient. When *α*_*A *_= *α*_*R *_= *α*, an iterative solution is used for *τ*_*X *_= *t*_*X *_- *t*_*C*_. Denoting Δ*β *= *β*_*A *_- *β*_*a *_- *α*_*A*_*K*_*A *_and $\Delta X=\sqrt{\beta_{a}/k}-(\beta_{r}/\alpha_{R})$, and assuming that *β*_*r*_/*α *<<*β*_*A*_*τ*_*X*_,

(44) $$\begin{array}{lll} \tau_{X} & = & \alpha^{-1}\ln[(\Delta \beta /\Delta X)\tau_{X}]\\ & = & \alpha^{-1}\ln[(\Delta \beta /\Delta X)\alpha^{-1}\ln[(\Delta \beta /\Delta X)\tau_{X}]\\ & = & \alpha^{-1}\ln[\Delta \beta /\alpha \Delta X]+\alpha^{-1}\ln\{\ln[(\Delta \beta /\Delta X)\tau_{X}]\}\\ & \approx & \alpha^{-1}\ln[\Delta \beta /\alpha \Delta X]+\alpha^{-1}\ln\{\ln[(\Delta \beta /\alpha \Delta X)\ln(\Delta \beta /\alpha \Delta X)]\}, \end{array}$$

where in the last equation the term *τ*_*X *_on the right-hand side has been replaced by its leading order value *α*^-1 ^ln [Δ*β*/*α*Δ*X*] to terminate the expansion.

### Time intervals

For convenience, time intervals are denoted with symbol *τ *as

(45) τK=tKτ′K=t′K−tKτC=tC−t′KτX=tX−tC.τtot=tX=τK+τ′K+τC+τX.

Simplified formulas for the intervals are obtained by using leading-order contributions based on biologically reasonable assumptions that threshold values (*K*_*A *_and *K*_*R*_) are small compared to the activated level of *A*, and that the fully activated production rate for *A*, *β*_*A *_is much larger than the baseline rate *β*_*a *_or the fully activated production rate *β*_*R *_of repressor, and that *β*_*r *_is 0.

In these limits,

(46) τK=[βA(n−1)]−1KAn(βa/k)1−n, or=(KA/βA)ln⁡(KA/βa/k) if n=1τ′K=αA−1(βA/βR)τC=KA/βAτX=αmin⁡−1ln⁡[βA/|αA−αR|βa/k]=α−1ln⁡[βA/αβa/k] if αA=αR.

Both *τ*_*K *_and *τ*_*C *_are fast, scaling as inverse production rates, while *τ'*_*K *_and *τ*_*X *_are slow, scaling as inverse decay rates. To compare with numeric simulation results, we break the entire cycle into two phases with times *τ*_1 _and *τ*_2_. The first phase begins at the start of the cycle with *A *= *R *and $\dot{A}$ > 0 and ends when *C *is at its maximum. The second phase begins when *C *is at its maximum and ends at the start of the next cycle:

(47) $$\begin{array}{lll} \tau_{1} & = & \tau_{K}+{\tau^{\prime }}_{K}+\tau_{C}\approx {\tau^{\prime }}_{K}\approx \alpha_{A}^{-1}\beta_{A}/\beta_{R}\\ \tau_{2} & = & \tau_{X}\approx \alpha_{\min}^{-1}\ln[\beta_{A}/|\alpha_{A}-\alpha_{R}|\sqrt{\beta_{a}/k}] \end{array}$$

### Energy cost

The energy cost of sustaining oscillations is estimated as the number of protein molecules synthesized per cycle, giving equal weight to activator and repressor proteins. During the first phase, activator proteins are synthesized at rate *β*_*A *_and repressor proteins at rate *β*_*R*_. During the second phase, synthesis rates are *β*_*a *_and *β*_*r*_. The cost per cycle is

(48) Cost = (*β*_*A *_+ *β*_*R*_)*τ*_1_/*τ*_tot _+ (*β*_*a *_+ *β*_*r*_)*τ*_2_/*τ*_tot_.

### Repressilator dynamics

The repressilator is a synthetic oscillator constructed from an odd cycle of negative feedback loops [6].

Again using a steady-state approximation for mRNA levels, the dynamics for the standard three-component symmetric continuous repressilator are

(49) $${\dot{P}}_{1}=\beta_{0}+(\beta_{1}-\beta_{0})K^{n}/(K^{n}+P_{i-1}^{n})-\alpha P_{i}$$

where *P*_*i *_represents one of *i *= 1 to 3 components, and *P*_*i*-1 _= *P*_3 _when *i *= 1.

Using the same approximations as for the hysteresis oscillator, the repressilator cycle starts when *P*_3 _falls just below *K*, with *P*_1 _at its baseline level *β*_0_/*α *and *P*_2 _roughly equal to (*β*_1_/*α*) - *K*. In this regime,

(50) $$\begin{array}{lll} P_{1}(t) & \approx & \left(\beta_{0}/\alpha )e^{-\alpha t}+(\beta_{1}/\alpha )(1-e^{-\alpha t}\right)\\ P_{2}(t) & \approx & -Ke^{-\alpha t}+(\beta_{1}/\alpha )\\ P_{3}(t) & \approx & Ke^{-\alpha t}+(\beta_{0}/\alpha )(1-e^{-\alpha t}). \end{array}$$

These dynamics continue until *P*_1_(*t*) = *K*, defined to occur after an interval *τ*_1_,

(51) *τ*_1 _= *α*^-1 ^ln [(*β*_1 _- *β*_0_)/(*β*_1 _- *αK*)] ≈ *K*/*β*_1_,

where the approximation holds when *β*_1_/*α *>> *K *> *β*_0_/*α*. At this point,

(52) $$\begin{array}{lll} P_{1}(\tau_{1}) & = & K\\ P_{2}(\tau_{1}) & = & (\beta_{1}/\alpha )-K(\beta_{1}-\alpha K)/(\beta_{1}-\beta_{0})\approx (\beta_{1}/\alpha )-K\\ P_{3}(\tau_{1}) & = & K(\beta_{1}-\alpha K)/(\beta_{1}-\beta_{0})+(\beta_{0}/\alpha )(\alpha K-\beta_{0})/(\beta_{1}-\beta_{0})\approx K \end{array}$$

with dynamics

(53) $$\begin{array}{lll} {\dot{P}}_{1} & = & \beta_{1}-\alpha P_{1}\\ {\dot{P}}_{2} & = & \beta_{0}-\alpha P_{2}\\ {\dot{P}}_{3} & = & \beta_{0}-\alpha P_{3}. \end{array}$$

These dynamics continue until time *τ*_1 _+ *τ*_2 _when *P*_2_(*τ*_1 _+ *τ*_2_) = *K*,

(54) *τ*_2 _= *α*^-1 ^ln [(*β*_1 _- *αK *- *β*_0_)/(*αK *- *β*_0_)] ≈ *α*^-1 ^ln(*β*_1_/*αK*).

This pattern repeats three times for a total period *τ*_tot _of

(55) *τ*_tot _= 3(*τ*_1 _+ *τ*_2_) = (3/*α*) ln [(*β*_1 _- *β*_0_)(*β*_1 _- *αK *- *β*_0_)/(*β*_1 _- *αK*)(*αK *- *β*_0_)].

### Energy cost

During the *τ*_+ _phase, components 1 and 2 are synthesized at rate *β*_1_, while component 3 is synthesized at rate *β*_0_. During the *τ *_ phase, only component 1 is synthesized at the higher rate. The energy consumption, in terms of proteins per cycle, is

(56) $$\begin{array}{lll} \text{Cost} & = & \beta_{0}+\beta_{1}+(\beta_{1}\tau_{1}+\beta_{0}\tau_{2})/\tau_{\text{tot}}\\ & \approx & \beta_{1}+2\beta_{0}+K/\tau_{2}. \end{array}$$

### Implementation and availability

The analytical and numerical equations are provided as R code as supplementary material released under the GNU Lesser General Public License version 3 [see Additional file 1]. Source code is also available from the author at .

## Authors' contributions

CK and VG assisted with the mathematical analysis and performed the numerical simulations. JSB conceived the modeling strategy, designed the study, and performed some of the simulations. All three participated in writing the manuscript.

## Supplementary Material

Additional file 1 **clocksim.r.** Source code for analytical and numerical simulations and plots. Click here for file
